# A case report of idiopathic spontaneous peritoneal and retroperitoneal hematoma of a pregnant woman

**DOI:** 10.1016/j.amsu.2021.102954

**Published:** 2021-10-19

**Authors:** Safaa Kachmar, Younes Oujidi, Zakaria Bouayed, Houssam Bkiyar, Brahim Housni

**Affiliations:** a Anesthesia and Resuscitation Department, MOHAMMED VI Teaching Hospital, OUJDA, Morocco; b Simulation Center, Faculty of Medicine and Pharmacy, Oujda Morocco

**Keywords:** Surgery, Laparotomy, Pregnancy, Hematoma, Peritoneum, Retroperitoneum

## Abstract

**Context:**

Peritoneal and retroperitoneal hematoma are usually secondary to trauma, an obstetrical pathology, an aneurysmal pathology or a tumorous pathology. A spontaneous idiopathic form remains rare, especially when it occurs to a pregnant woman, which makes the clinical and etiological diagnosis difficult, as well as the therapeutic management both the mother and the fetus.

**Case report:**

We report the case of a spontaneous idiopathic hemoperitoneum and hemoretroperitoneum of a 26-year-old woman, pregnant (30th week of amenorrhea), presenting a hemodynamic instability and a clinical acute surgical abdomen. No secondary cause was identified during exploratory laparotomy, neither through imaging. The therapeutic management relied on hemodynamic stabilization after exploratory laparotomy.

**Conclusion:**

Idiopathic spontaneous peritoneal and retroperitoneal hematoma -in the presence of several differential diagnoses-remain an extremely rare entity to evoke in front of an acute surgical abdomen in a pregnant woman.

## Highlights

• Idiopathic spontaneous hemoperitoneum and hemoretroperitoneum is a rare condition, with only few cases reported in literature.
• The occurrence of an idiopathic spontaneous hemoperitoneum and hemoretroperitoneum in pregnant women is a serious condition engaging both the patient's and the fetus's prognosis.
• Management consist of a thorough investigation in search of an etiological process before retaining the diagnosis, as well as a hemostatic procedure.

## 1 Introduction

Hemoperitoneum and hemoretroperitoneum are usually caused by trauma, ruptured spleen aneurysm, ectopic pregnancy, ovarian follicle bleeding, uterine rupture, retro-placental hematoma, or a liver tumor.

Idiopathic spontaneous hemoperitoneum and hemoretroperitoneum are a very rare entity, characterized by a diffuse abdominal pain associated with a cardiovascular instability.

We discuss a case of an idiopathic spontaneous hemoperitoneum and hem retroperitoneum of a 26-year-old woman, pregnant (30th week of amenorrhea), who presented a hemorrhagic shock associated to an acute surgical abdomen, with no etiology found despite careful surgical exploration and oriented radiological examinations.

## 2 Case report

A 26-year-old woman, with no prior medical history, primiparous, pregnant at her 30th week of amenorrhea who consulted the emergency room for extreme asthenia. She did not report any notion of trauma, vasculitis, or coagulopathy. Clinically, the patient was shocked with a low blood pressure (83/51 mmHg), tachycardia (121 bpm), oliguria, generalized pallor, and confusion (Glasgow Coma Scale 13/15) without externalized bleeding (including no hematuria or utero-vaginal bleeding). Clinical examination revealed a diffused abdominal guarding with right iliac fossa and hypogastric sensitivity with negative fetal heart sounds.

Initial management consisted of stabilizing the patient by perfusing 500 ml of Hydroxyethylamidon followed by transfusing 6 units of packed red blood cells, 6 units of fresh frozen plasma, infusing 3g of fibrinogen, administration of 1g of tranexamic acid, 4g of calcium and 5 mg of norepinephrine with invasive monitoring of blood pressure.

Blood work revealed a hemoglobinemia at 8.5g/dl, a platelet count at 252000/mm^3^, a prothrombin at 68%, and fibrinogen at 2.1 g/L. liver and kidney function tests were strictly normal. An abdominopelvic ultrasound showed a very considerable hemoperitoneum and hemoretroperitoneum.

**Fig. 1:**
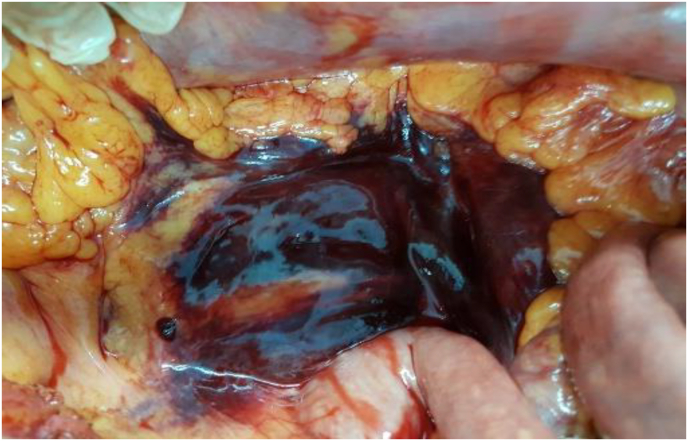
Perioperative image showing the retroperitoneal hematoma.

**Fig. 2:**
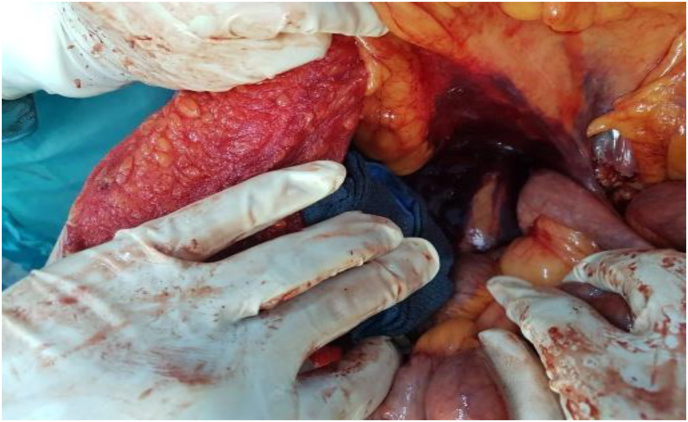
Perioperative image showing the retroperitoneal hematoma.

**Fig. 3:**
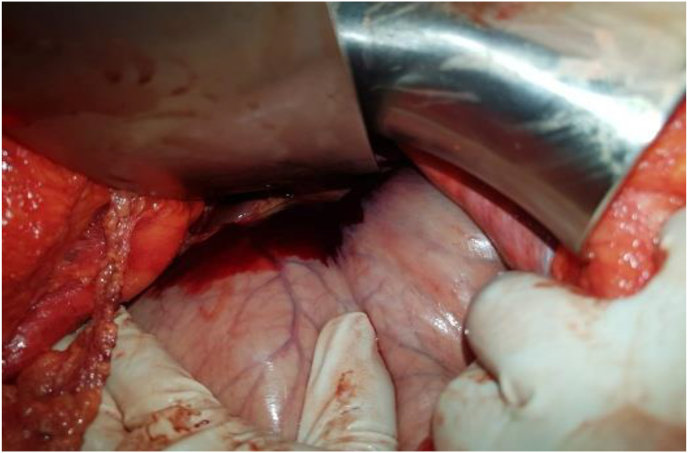
Perioperative image showing the peritoneal hematoma.

**Fig. 4:**
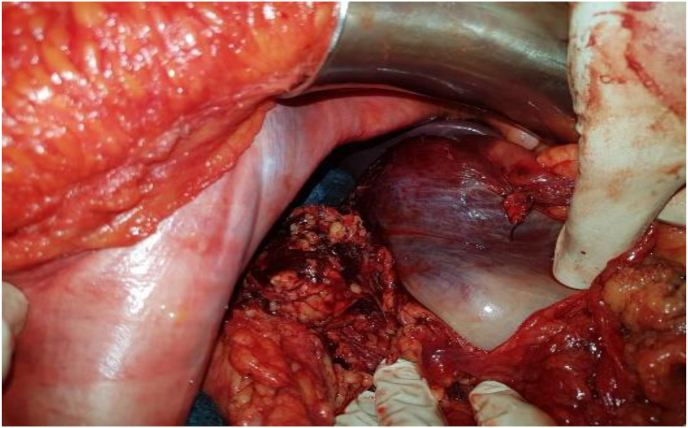
Perioperative image showing the peritoneal hematoma.

The patient was sent to the operating room urgently. The exploration revealed a huge expansive retroperitoneal hematoma (Fig. 1, Fig. 2) and a large hemoperitoneum (Fig. 3, Fig. 4).

**Fig. 5:**
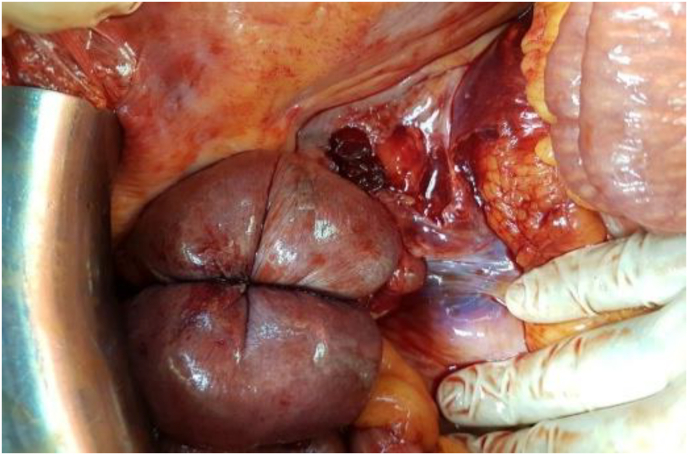
Triple ligature and B-LYNCH of the uterus.

The gesture involved a fetal extraction, with a triple ligature and B-LYNCH of the uterus because it was atonic (Fig. 5). Then, splenectomy and hemostasis of the hem retroperitoneum by temporary tamponade, drainage and closure.

**Fig. 6:**
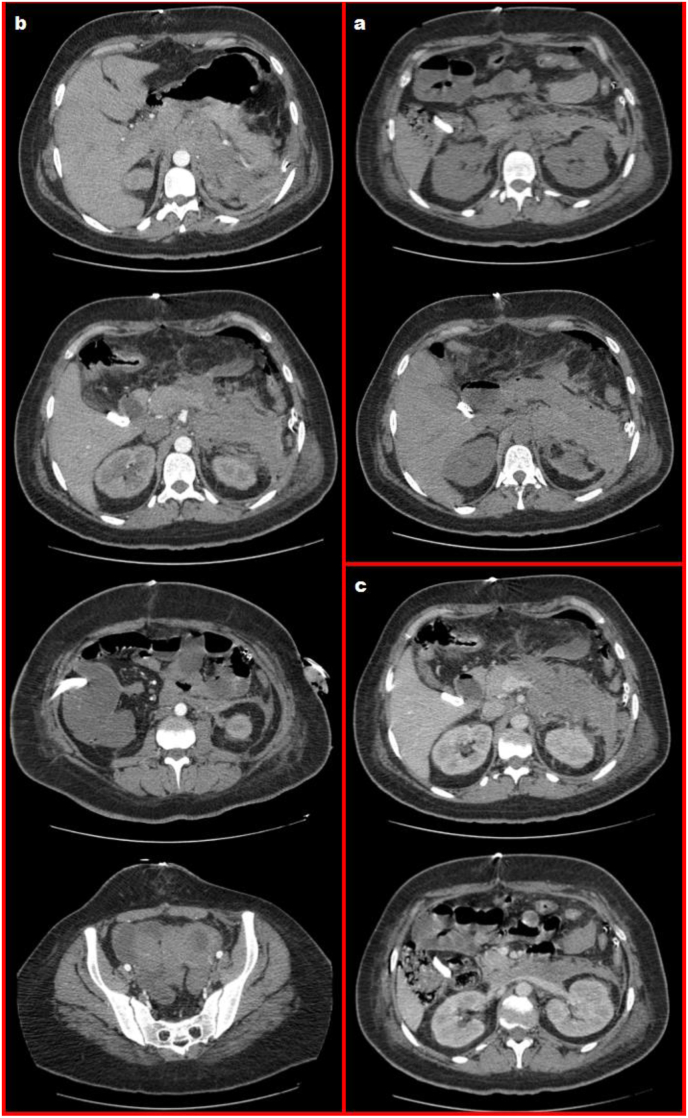
Axial C- (a), C+ arterial phase (b), and C+ venous phase (c) abdominal CT sequences showing: Spontaneously Hyper intense collection in the bolsa omental facing the pancreatic tail without contrast media extravasation in C+ sequences, Infiltration of the mesenteric fat facing the pancreatic body spontaneously hyper intense, Redon drain within the (empty) splenic lodge without contrast media extravasation in C+ sequences, Delbet drain within the hepatic hilum, Intra-peritoneal effusion of low abundance within the Morrison's pouch, the left peri-colic gutter, and in the pelvis, Poly-lobed gravid uterus.

After surgery, an injected abdominopelvic CT showed a regression of the retroperitoneal hematoma with no signs of recurrence, and persisting peritoneal hematoma (Fig. 6).

## 3 Discussion

Hemoperitoneum and hemoretroperitoneum during pregnancy are often fatal for the fetus and remain a rare entity shadowed by many differential diagnoses. Among others, retroplacental hematoma, uterine rupture, rupture of a splenic artery aneurysm, placenta percreta, spontaneous rupture of hepatic hemangioma, rupture of utero-ovarian veins, or hemorrhagic cysts with a pre-existing coagulopathy.

According to a literature review, other cases of idiopathic spontaneous retroperitoneal hematoma have been reported [[1], [2], [3]]. It is a relatively rare pathology that clinically manifests by an acute surgical abdomen [4]. For pregnant women, the pathophysiology would be the gravid capillary fragility. A rupture of the small vessels can cause a large hematoma [5]. The etiology is generally found during exploratory laparotomy or during radiological investigations after surgery [6]. Similarly in our case, the etiology was not found despite ultrasound, and tomography however, the diagnosis was retained during laparotomy, backed by enhanced computed tomography after surgery.

Therapeutic management usually starts with hemodynamic stabilization by infusion of fluids, transfusion of blood derivatives, administration of vasoactive drugs and correction of coagulopathy. After that, hemostasis should be maintained. Indeed, endovascular treatment has an important role, but should be recommended only for stable patients. On the other hand, laparotomy should be reserved in case of failure or non-availability of endovascular treatment and for hemodynamically unstable patients, as is the case with this patient. However, the best approach can be difficult to assess [7].

In obstetric care, it would depend on gestational age. Indeed, when the age of the pregnancy is less than six months, a therapeutic abstention is essential with strict supervision of the mother and the fetus. In case of fetus death, therapeutic management will proceed to evacuation. But if the pregnancy continues, a caesarean section is recommended at term [5]. If the pregnancy is older than six months old, a caesarean section is preferable for both the mother and the fetus [5]. For our patient, as described, an evacuation of the dead fetus was carried out urgently for the sake of the mother's survival.

## 4 Conclusion

Idiopathic spontaneous peritoneal and retroperitoneal hematoma, in the presence of several differential diagnoses, remain an extremely rare entity to consider in front of an acute surgical abdomen presented by a pregnant woman. The diagnosis is difficult, affecting the therapeutic management; which should be rapid and codified to ensure maternal and fetal rescue.

This paper has been reported in line with the SCARE 2020 criteria [8].

## Patient consent

Written informed consent was obtained from the patient for publication of this case report and accompanying images. A copy of the written consent is available for review by the Editor-in-Chief of this journal on request.

## Provenance and peer review

Not commissioned, externally peer reviewed.

## Ethical approval

This is a care report; therefore ethical approval isn't needed.

## Sources of funding

Our case report did not receive any funding.

## Author contribution

SAFAA KACHMAR: participation in the management of the patient, redaction of the article, bibliographic research, submission in your journal. YOUNES OUJIDI: participation in the management of the patient, bibliographic research. ZAKARIA BOUAYED: contributor, submission to the journal. HOUSSAM BKIYAR: correction of the article before submission, orientation of the article's redaction and bibliographic research. BRAHIM HOUSNI: correction of the article before submission, orientation of the article's redaction and bibliographic research.

## Registration of research studies

Name of the registry:
Unique Identifying number or registration ID:
Hyperlink to your specific registration (must be publicly accessible and will be checked):

## Guarantor

Dr. SAFAA KACHMAR.

## Declaration of competing interest

There are no conflicts of interest.

## References

[1] Vionnet M., Rostan O. (2003). [Idiopathic spontaneous hemoperitoneum]. Swiss Surg..

[2] Matsuyama T., Nakatsuka H., Asahara T., Dohi K. (1986). Idiopathic retroperitoneal hematoma presenting as acute abdomen. Hiroshima J. Med. Sci..

[3] Ploteau S., Podevin J., Frampas E. (2003). Hémopéritoine « idiopathique » de la femme enceinte : à propos de deux cas. J Gynécologie Obs Biol la Reprod.

[4] Brown C.F., Mashini I.S., Turner W.A., Gallup D.G. (1986). Retroperitoneal hematoma: an unusual complication of cold knife conization of the cervix. Obstet. Gynecol..

[5] Redźko S., Przepieść J., Urban J. (2003). [Retroperitoneal hematoma during pregnancy--diagnostic dilemma]. Ginekol. Pol..

[6] Scialpi M., Scaglione M., Angelelli G. (2004). Emergencies in the retroperitoneum: assessment of spread of disease by helical CT. Eur. J. Radiol..

[7] Chan Y.C., Morales J.P., Reidy J.F., Taylor P.R. (2008). Management of spontaneous and iatrogenic retroperitoneal haemorrhage: conservative management, endovascular intervention or open surgery?. Int. J. Clin. Pract..

[8] Agha R.A., Franchi T., Sohrabi C., Mathew G., Kerwan A. (2020). The SCARE 2020 guideline: updating consensus surgical CAse REport (SCARE) guidelines. Int. J. Surg..

